# Study protocol for the family empowerment program: a randomized waitlist-controlled trial to evaluate the effectiveness of online Community Reinforcement and Family Training (CRAFT) on the wellbeing of family members with a relative experiencing substance dependence and mental illness

**DOI:** 10.1186/s12888-023-05487-0

**Published:** 2024-01-10

**Authors:** Julaine Allan, Nicole Snowdon, Subash Thapa, Kedir Y. Ahmed

**Affiliations:** Rural Health Research Institute, Charles Sturt University, Leeds Parade, Orange, NSW 2800 Australia

**Keywords:** Rural, Substance disorder, Online therapy, Community reinforcement and family training, Family, Mental health

## Abstract

**Background:**

Systematic reviews consistently show that family-focused interventions are effective at improving substance treatment engagement and outcomes across the lifespan. Yet, Australian substance use treatment services rarely incorporate family members and concerned significant others. Testing of family focussed interventions in the Australian context is required.

**Methods:**

The trial is a randomized wait-list control trial assessing the effectiveness, feasibility and acceptability of online CRAFT with a parallel group. Participants will be randomised to receive either online CRAFT or to a wait-list control group who are provided with CRAFT related reading material during the waiting period. Outcomes will be assessed at baseline and then at 6- and 15-weeks post baseline. The primary outcome will be improved wellbeing of participating family members. The trial reporting will comply with SPIRIT guidelines.

**Discussion:**

This study will focus on people living in rural areas. Substance treatment programs are limited in rural Australia. The provision of the Family Empowerment Program (CRAFT) online should make family focused substance treatment support accessible and attainable for the first time in rural areas. The outcomes of this trial could have meaningful implications for the future funding and support of family focused substance treatment services that are inclusive of people with mental health conditions.

**Trial registration:**

ANZCTR, ACTRN12623000796684p, Registered 26 July 2023. Prospectively registered with protocol version 3.

**Supplementary Information:**

The online version contains supplementary material available at 10.1186/s12888-023-05487-0.

## Background

Substance use is associated with poor outcomes for individuals with mental illness because it impedes treatment engagement and is associated with more severe psychiatric symptoms [1]. Systematic reviews consistently show that family-focused interventions are effective at improving substance treatment engagement and outcomes across the lifespan [2, 3]. Yet, substance treatment services rarely incorporate family members and concerned significant others [4]. Integrated treatment, care and support for people living with mental illness and substance use is the goal of policy and practice in Australia and internationally. However, while the direction has been given by state and national governments to “do” integrated care, there are no frameworks or guidelines about what to do [5]. Ways to address problematic substance use of people with a mental illness are needed and family members are best placed to provide support.

According to the Australian Institute of Health and Welfare [AIHW], 45% of all Australians aged 16 to 85 years — 8.7 million people — will experience mental illness at some point in their life [6]. The most common conditions are anxiety, depression and alcohol dependence. Mental illness and substance misuse are the second largest contributor (23%) of the non-fatal burden of disease in Australia with $9.9 billion being spent on mental health in 2017–18 [7]. Given the prevalence and social and economic costs of mental illness including substance dependence, access and effectiveness of mental health services is crucial.

Harmful substance use and related deaths are more prevalent in rural Australia compared to cities [8]. However, substance treatment services are mostly located in large cities rather than in regional or rural areas [9]. Further, services can meet only one third of the demand for treatment [9]. Few of these services provide support to family members. Only 7% of the 139,300 clients receiving treatment in 2021 were family or friends of people with a substance problem [9].

In Australia, one in three individuals with a substance use disorder also has a mental health disorder [6]. Two or more conditions are so common among those experiencing mental health or substance use problems that “comorbidity is seen as the rule rather than the exception” [10]. Further, comorbidity results in a greater care burden on the family and increased family conflict compared to single disorders [11–13]. However, very often the presence of co-occurring conditions excludes people from participating in clinical trials and the efficacy of interventions for people with more than one condition is unknown [14].

The aim of the current study is to implement and evaluate Community Reinforcement and Family Training (CRAFT) delivered online by trained counsellors to people living in rural Australia. CRAFT is an evidence-based approach that helps families to reduce a relative’s substance use, engage in treatment, and improve family wellbeing through structured, personalised training and support [2, 15–17]). However, CRAFT is not available in Australia and has limited evidence for mental illness and substance use combined [12]. Derived from Cognitive Behavioural Therapy (CBT) and Motivational Interviewing (MI), the aims of CRAFT are to teach family members how to remove positive reinforcement for problematic substance use behaviour effectively and safely, increase positive reinforcement for non-using behaviour, and help the person with problematic substance use to enter, or be retained in, treatment [18]. In addition, CRAFT aims to improve family members’ social and emotional wellbeing.

In this iteration, CRAFT will be delivered as The Family Empowerment Program, emphasizing family members as the primary target group for the study and aiming to establish CRAFT as a family-focused therapy program in Australia. The study will generate the first Australian outcome data, which will complement the USA outcome data [12] and the Australian adaptation and acceptability data from the team’s previous work [16, 19]. This research project can inform future health system policy on the provision of virtual care for rural Australian families of people with substance dependence and mental illness.

### Objectives of the study

This trial will determine the effectiveness of an online delivered CRAFT counselling intervention for improving the well-being of family members with a loved one experiencing substance problems with and without co-occurring mental health problems. It is hypothesised that CRAFT implemented online by accredited therapists will be more beneficial than CRAFT self-help information provided to the wait-list group. Compared to the wait-list control group, the CRAFT group will show significant decrease in self-reported levels of depression, anxiety and stress and significant increase in life satisfaction and that these improvements will be maintained over a three-month period post intervention.

It is hypothesized that 1) improvements in depression, anxiety and stress scores as a function of CRAFT relative to the control group will be mediated by: a) acceptability of receiving support online and b) ease of access. Given the lack of information regarding implementation parameters of substance use interventions for families of people with a mental health condition, qualitative methods will explore the experiences of study participants as well as clinicians delivering the program. The study has the following specific aims:

- Aim 1: determine the effectiveness of an online delivered CRAFT counselling intervention on on self-reported levels of depression, anxiety, stress and life satisfaction
- Aim 2: qualitatively examine the paricipants’ experiences about the acceptability of receiving support online
- Aim 3: qualitatively examine the clinicians’ experiences of delivering the program and feasibility

## Methods

### Study design

**Fig. 1:**
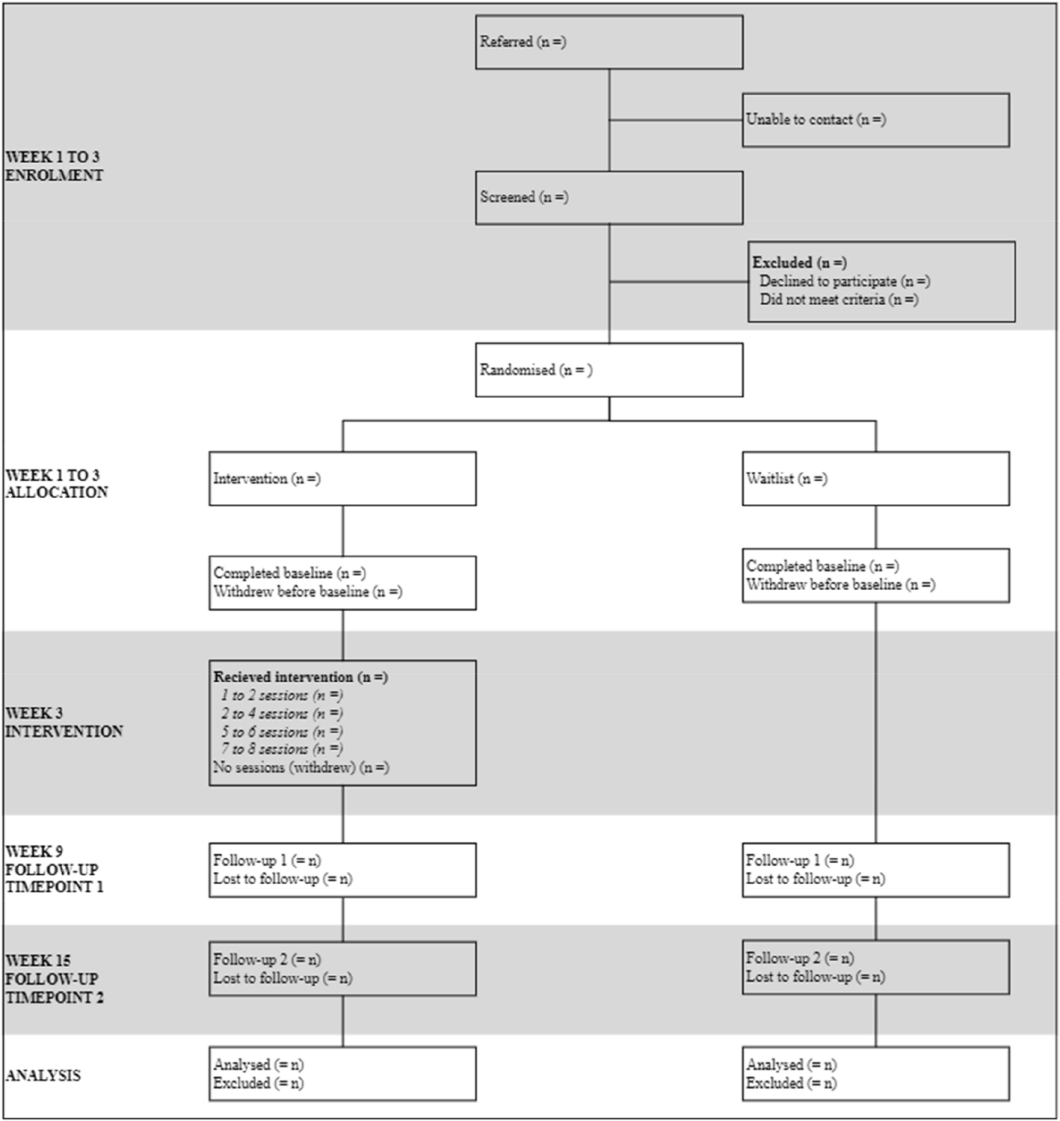
CONSORT diagram

This is a randomized wait-list control trial that will evaluate the effectiveness, feasibility and acceptability of online CRAFT with a parallel group. Participants will be randomised to receive either online CRAFT for 6 sessions over 6 to 10 weeks or to a wait-list control group who are provided with CRAFT related reading materials during the waiting period. Outcomes will be assessed at baseline and then at 6- and 15-weeks post baseline. Outcome measures will be completed online independently of researchers or clinicians. The trial reporting will comply with CONSORT guidelines. Figure 1 provides an overview of the trial recruitment and process.

### Eligibility criteria

Eligible participants will be 18 years or older with a relative or loved one who has a substance problem with or without another mental health condition. Participants must live in a rural area (Modified Monash Model 2–7), have a minimum of weekly contact with their loved one, willing to attend at 6 online counselling sessions, access to a computer with internet or mobile phone with video conferencing capabilities and able to provide informed consent. Exclusion criteria includes domestic and family violence from the person with substance dependence to the participating family member and/or current participation in family support/therapy programs related to substance use.

### Recruitment and follow-up procedures

Participants will be recruited from any rural areas in Australia by advertisements through healthcare and substance treatment agencies and health advice internet sites, in local newspapers and radio stations and paid geo-located (rural) social media advertisements (see Supplementary Material). The advertisement will include information about CRAFT, highlighting that the program’s goals are to improve the participants’ mental health and increase the likelihood of their relative’s engagement in treatment. Individuals who are interested in participating will register on a secure website or by contacting the study coordinator.

Those who are registered will be contacted within 48 h and invited to complete a short telephone screen using the inclusion and exclusion criteria. If eligible, the study information and process will be described, and verbal consent will be gained. Following this, participants are randomised to either the waitlist or intervention group, and a first initial appointment is organised with an in-community CRAFT practitioner. Participants will then be sent a link to the written consent form and outcome measures.

On average, the information and screening process will take approximately 20–30 min to complete. Once the consent is signed, the participant can access the online baseline measures for completion. If the measures are not completed, SMS reminders will be sent after 2 days. If the participant still does not complete the measures, they will be contacted by phone to confirm their intent to join the study and to ensure that they can access the survey. Participants who complete all three surveys (baseline, 6 and 15 weeks) will receive $120AUD in gift card for their time and participation. Individuals who have not responded to the initial call or have not completed the follow-up measures will be contacted on a weekly basis by both phone and SMS for up to 4 weeks after the follow-up is due.

### Randomisation and blinding

The randomisation procedure for this project will be conducted by an independent researcher (KYA) using STATA software (Version 18 MP) and with the 'randomizr' package. This researcher will not be involved in other aspects of project implementation, such as CRAFT delivery or data collection. The researcher (NS) who recruits the participants will have no access to the order of allocation. All participants will be allocated an identification number and, before completing their baseline measures, will be randomly allocated to either the CRAFT group or the wait list control group. Individuals randomized to the control group will be assigned a 6-week wait time.

All assessments are conducted online, thus minimizing the possibility of researcher bias. Analysis of results will be blinded to the clinicians delivering CRAFT. The clinicians will be provided with contact information of consenting participants and they will deliver the program as scheduled without knowing which group the person is allocated to. Trial participants will be informed their start date for the intervention as 1. as soon as possible (Group A—within 7 days) or 2. within 6 weeks (Group B—WLC) without being informed which group they are in. The need for unblinding is not anticipated.

### Measures

The outcome measures will take participants approximately 20 min to complete (Table 1).

**Table 1:** Baseline (BL) and follow-up measures assessment schedule

Instrument	Domain	BL	6 weeks	15 weeks
DASS 21	Psychological wellbeing	x	x	x
Satisfaction with Life Scale (SWLS)	Quality of life	x	x	x
The Flourishing Scale (FS)	Psychological wellbeing	x	x	x
Brief-COPE	Stress and distress management	x	x	x
Use of online delivery methods (session summary sheet)	Delivery experience		x	
Number and length of sessions attended Sessions delivered (Session summary sheet)	Feasibility Fidelity		x	
Experience interviews	Acceptability			x

### Demographics

Age, gender, ethnicity, living arrangements, financial support, years of education, relationship status and relationship to the relative, mental health diagnosis of relative will be collected.

### Depression, Anxiety, and Stress Scale (DASS-21)

The DASS-21 [20] is a 21-item self-report questionnaire designed to measure the domains of depression, anxiety and stress. Each subscale contains 7 items rated on a four-point Likert scale. Higher scores indicate more severe symptoms. The DASS-21 is reported to have high internal consistency for each of the subscales (depression, *r* = 0.88; anxiety, *r* = 0.82; stress *r* = 0.90), and the total scale (*r* = 0.93) [21].

### Satisfaction with Life Scale (SWLS)

This is a widely used 5-item self-report measure of life satisfaction [22]. The scale includes a cognitive judgment component and an emotional component. The SWLS uses a 7-point Likert scale from 1 (strongly disagree) to 7 (strongly agree). The range of possible scores is from minimal satisfaction with life (5) to very high satisfaction with life [22]. The SWLS has demonstrated convergent validity with other self-report measures of life satisfaction, including the Philadelphia Geriatric Center Morale Scale [23]. The SWLS appears to identify a single life satisfaction factor. Internal consistency is good with a Cronbach’s alpha coefficient of 0.83 [23].

### The Flourishing Scale (FS)

The FS is an eight item self-report measure of psychological well-being [24]. The scale concepts include feelings of competence, positive relationships, and purpose in life. Respondents rate on a 7-point Likert scale from strong disagreement to strong agreement with statements. Scores range from 8 to 56 and high scores indicate positive self report of wellbeing [25]. The scale developers report strong correlations with other psychological well-being scales and good internal consistency with Cronbach’s alpha coefficient of 0.87 [24].

### Brief-COPE

The Brief-COPE is a 28 item self-report questionnaire designed to measure effective and ineffective ways to cope with a stressful life event [26]. “Coping” is defined broadly as an individual’s effort used to minimise distress associated with problems in life. The scale identifies a person’s primary coping styles via scores on fourteen factors across three subscales: Problem-Focussed Coping; Emotion-Focussed Coping and Avoidant Coping [26]. Effective coping strategies are linked to good self-esteem, lower perceived stress, and lower psychological distress, whereas less functional strategies (for example denial, substance use and/or self-blame) are linked to poor self-esteem, to higher perceived stress, and to psychological distress [27]. The 14-factor structure of the Brief COPE has good psychometric properties [28].

#### Treatment fidelity and feasibility

All counsellors delivering CRAFT for The Family Empowerment Program will be required to be certified as CRAFT Therapists following standardised procedures established by Robert Meyers [18]. Each counsellor will complete a session summary sheet for each client (see Supplementary Material) for each session. The summary sheet will record which CRAFT procedures were delivered and also identify any problems experienced with the online delivery.

#### Treatment acceptability

Treatment satisfaction will be evaluated via individual interview with participants at the 15-week follow-up. All participants who have completed at least one session of the program will be invited to an interview about their experiences with the program and any related benefits and challenges. Acceptability will be further assessed via individual interviews with clincians who have delivered the program. These interviews will be conducted when recruitment has finished. All clinicians who delivered at least one session will be invited to participate in an interview with the aim of capturing challenges for those who did not regularly deliver the program.

#### Sample size calculation

The study's sample size is determined using data from a previously published cluster randomized trial [29]. Taking into account an outcome of treatment engagement rate of 29% for the online FEP intervention group and 15% for the control group, along with a 5% level of significance (α) and 80% power, the calculated sample size is 216 (with 108 participants in each group). To account for a conservatively estimated attrition rate of 20% during follow-up, a total of 260 participants will be recruited.

#### Statistical analysis

This study will employ a three-stage analytical approach. In the first stage, baseline characteristics of study participants will be identified using descriptive statistics and standard deviations [30]. The second stage involves bivariable analysis using the chi-square test for categorical variables and the t-test for continuous variables, including the estimation of differences in proportion and mean (difference-in-difference) between intervention groups [30]. To assess the intervention's effects on primary outcomes, mixed effect regression modelling will be applied. Intervention effects will be quantified using mean differences (MD) for continuous variables and relative risks (RR) for categorical variables, along with their corresponding 95% confidence intervals (CI) [31]. The primary results will be based on adjusted effect measures, and all analyses will adhere to the intention-to-treat (ITT) principle [32]. STATA version 18 will be used for the analyses, and statistical significance will be declared by *p*-values < 0.05.

#### Trial duration

The study has commenced in October 2023 and the recruitment will continue until November 2024. A preliminary analysis will be conducted after 20 participants have completed the program to assess for futlity. If no improvements in participant well-being are found, or wellbeing deteriorates or adverse events are reported the trial will be discontinued. This decision will be made by the research team collaboratively.

#### Ethics and data monitoring

Ethical approval has been received from the Charles Sturt University Human Research Ethics Committee (approval number H23769). The establishment of a Data Monitoring Committee is not required for this study because the trial is small scale and of short duration. The population is not particularly vulnerable and the intervention has a known safety profile. Risk management processes are established including reporting pathways for adverse events to the responsible Human Research Ethics Committee.

## Discussion

This study describes a randomised controlled trial of online delivery of CRAFT, a therapy program for family members concerned about a relative’s substance use. In this program family members whose relative also experiences a mental health condition will be included in the study. There are few substance treatment programs designed for family members even though the family are frequently the ones who provide care and support to people with substance dependence, bearing the brunt of associated health, financial and relationship difficulties [3, 4]. While people with substance problems are not the focus of this study, it does specifically include family members whose relative has both substance dependence and a mental health condition. A similar study [12] found that family members did not know how to talk about substance use with their relative who had a mental illness, even though the substance use was having critically negative effects on their psychiatric treatment. The Family Empowerment Program (CRAFT) offers a structured and tested way to communicate effectively about substance use and its effects, potentially improving mental health treatment for the relative. Any impact in this area will be picked up through the qualitative interviews with participants and will identify areas for future research.

This study will focus on people living in rural areas. Substance treatment programs are limited in rural Australia [8, 9]. The provision of the Family Empowerment Program (CRAFT) online should make family focused substance treatment support accessible and attainable for the first time in many rural areas. The delivery of the program via trained and accredited counsellors assures the fidelity to the CRAFT program and a quality service. These aspects of program delivery will be carefully evaluated through fidelity checklists and follow-up interviews with participants. The outcomes of this trial will have meaningful implications for the future funding and support of family focused substance treatment services that are inclusive of people with mental health conditions.

### Supplementary Information

**Additional file 1.**

**Additional file 2.**

**Additional file 3.**

## Data Availability

No datasets were generated or analysed during the current study.
