# Supplementary material for: Study protocol for the family empowerment program: a randomized waitlist-controlled trial to evaluate the effectiveness of online Community Reinforcement and Family Training (CRAFT) on the wellbeing of family members with a relative experiencing substance dependence and mental illness
Source: BMC Psychiatry. 2024 Jan 10;24:43. doi: 10.1186/s12888-023-05487-0 (PMC10782775; doi:10.1186/s12888-023-05487-0)
Supplement: Supplementary file 1 — Additional file 1. [file 12888_2023_5487_MOESM1_ESM.docx]

**Efficacy, feasibility and acceptability of the Online Family Empowerment Program.**

**What is this about?**

**
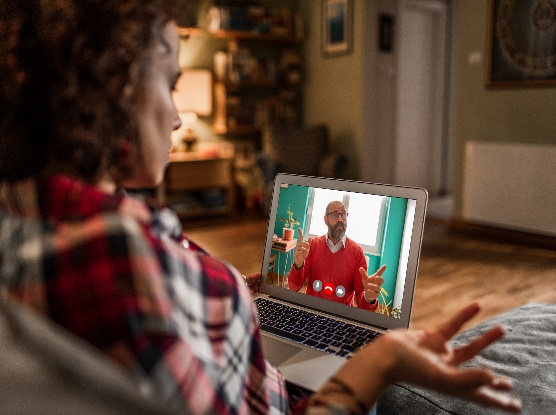
**This project is researching people’s experiences with a counselling program for family members of people with substance problems and mental health conditions. The Family Empowerment Program is a practical program of talking, planning and problem solving that improves a family member’s wellbeing and encourages their relative into drug and alcohol treatment. The project will provide participants with 6 online support sessions delivered by trained psychologists and social workers. The program has worked well in the United States but has not been available in Australia until now. We want to know if it works for rural Australians.

The study is being conducted by Dr Julaine Allan, Ms Nicole Snowdon, Dr Kedir Ahmed and Dr Subash Thapa - all staff from Charles Sturt University; Professor Anthony Shakeshaft from the University of QLD and Dr Sara Farnbach from the National Drug and Alcohol Research Centre at UNSW are also part of the team. The research team will also include a PhD student [Heidi Gray] who will assist with data collection.

**What will I do?**

Participation in this project involves attending 6 free online counselling sessions and completing an online survey about your day-to-day wellbeing, stress, anxiety, depression and coping skills. You will be sent the survey on 3 occasions - before the program starts; at the end of the 6 weeks and three months after you have finished the program. Each survey will take about 30 minutes to complete, and you will be paid $120 for completing all 3. We also want to interview people after the program about their experiences of participating in the sessions. If you stop attending the sessions before they are finished, we will still contact you for the data collection and interview because everybody’s information is important for the data collection even if the Family Empowerment program wasn’t right for you.

If you are interested in receiving the Family Empowerment Program a member of the research team will talk through the project with you to answer any questions and will conduct a short interview to make sure you are eligible for the program. You will be asked to sign a formal consent form. If you consent to participate your contact details will be given to a Family Empowerment Program counsellor who will arrange an appointment time with you. Some people will be given appointments within a week, others will wait up to 6 weeks. This is so we can compare the results for different time periods. You will meet with the same counsellor online for an hour, once a week for 6 weeks.

**How do I know if I am eligible?**

To participate in this project, you need to meet the following criteria:

- Have a relative with a substance problem who you are in regular contact with even if you don’t live with them
- Live in a rural or remote area
- Be aged 18 years or older
- Have a phone or computer with internet access for online video calls
- Able to attend 6 one-hour sessions over 6 to 8 weeks
- Speak English
- Not be attending a substance use family support program
- If your relative is violent or controlling towards you, you cannot participate in the study because changing patterns of behaviour can provoke more violence. If family violence is a concern the study coordinator will discuss support options with you during the screening session.

**You can withdraw from the study.**

Being involved in the study is voluntary and you can leave the project at any time. If you change your mind about participation in the research, you will not be contacted for further data collection. However, any data already collected will be used in the study.

**
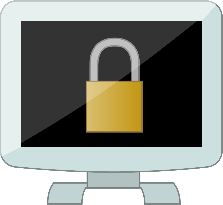
Your information is private**

Your information will be kept private through a unique code that is linked to your data rather than your name. When we write anything about the project, we will use this code. Everything you answer is confidential (unless you tell a researcher or counsellor something that is illegal or puts you at risk and they must report it by law). The research data and the counselling sessions are separate. The counsellors will not see your private information collected in the survey or interview and the research team will not have access to the information you share with the counsellor. The counsellors will only tell the research team if you attended scheduled sessions or not, or if they must report a crime or risk but not the details of that event. We will keep all the study documents and information in locked computer storage at Charles Sturt University for five years and then it will be destroyed. We will report the study findings in public reports, papers in openly accessible academic journals and at conferences. The data will not be used in other studies.

**
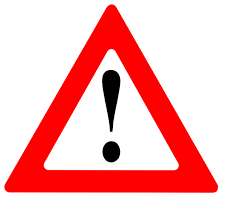
What are the possible risks of participating in this project?**

Talking about family problems and substance use may cause emotional distress and discomfort. If you do become distressed your counsellor will provide support during the session. You do not have to continue in the study if you find it too distressing. If you need help with something not related to the program (medical care for example) you will be provided with contact details for support services close to where you live. You may want to continue working with your counsellor after the free sessions have finished. You will need to discuss this with your counsellor including the costs and make your own arrangements for ongoing support. Your counsellor will raise this with you before the program ends.

**
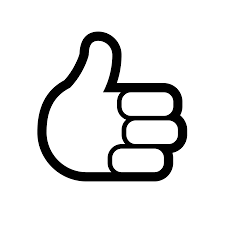
Benefits of the research**

The research will contribute to our understanding of ways we can deliver effective online support programs to reduce distress and enhance wellbeing of those impacted by someone else’s substance use, especially if they have a mental illness. The findings of this research will be able to be scaled up and adopted by healthcare services.


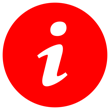
**How do I find out the results of the study?**

If you like, we can send you a brief report telling you about what we found out when the study is finished in June 2025. If you want this information, there will be a place to record your email address on the consent form.

**
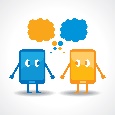
Who can I talk to about this project?**

You can contact the project leader if you have questions: **Associate Professor Julaine Allan** Rural Health Research Institute, Charles Sturt University Telephone: 02 6365 7119 Email: [juallan@csu.edu.au](mailto:juallan@csu.edu.au)

Charles Sturt University Human Research Ethics Committee has approved this project (no. ). If you have any complaints or reservations about the ethical conduct of this project, you may contact the Committee through the Research Integrity Unit via the following contact details:

The Presiding Officer

Human Research Ethics Committee

Research Integrity Unit

Locked Bag 588

Wagga Wagga NSW 2678

Phone: (02) 6933 4213

Email: [ethics@csu.edu.au](mailto:ethics@csu.edu.au)

Any issues you raise will be treated in confidence and investigated thoroughly, and you will be informed of the outcome.

This study has received funding from the Commonwealth Department of Health and Aging.


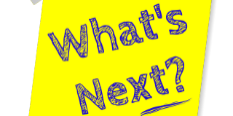


**What’s next?** Thank you for reading or listening to this information. If you agree to take part, we need you to complete the consent form with a researcher and then we will tell you when you receive the first survey and when your counsellor will contact you.

**Informed Consent**

**Project title:** Efficacy, feasibility and acceptability of the Online Family Empowerment Program.

**Investigators:** Dr Julaine Allan, Ms Nicole Snowdon, Dr Kedir Ahmed, Dr Subash Thapa, Professor Anthony Shakeshaft, Dr Sara Farnbach, Ms Heidi Gray

**Purpose:** This project is researching people’s experiences with a counselling program for family members of people with substance problems and mental health conditions.

**Name of participant:**

_________________________________________________________________________________

1. I consent to participate in this project, the details of which have been explained to me, and I have been provided with a written information sheet to keep.

2. I understand that after I sign and return this consent form it will be retained by the researcher.

3. I understand that my participation will involve:

(a) completing an online survey at 3 separate times that measures psychological wellbeing, stress, depression, anxiety and coping skills

(b) participating in 6 online sessions of the Family Empowerment Program with a trained counsellor

(c) an audio-recorded interview about my experience of the Family Empowerment Program

4. I agree that the researcher may use the results as described in the participant information sheet.

**Participant signature: Date:**

**__________________________________________________________________________________**

I would like to receive a summary of the study results when it is finished. Please send them to my email address ________________________________________
