# Supplementary figures and images for: Study protocol for the family empowerment program: a randomized waitlist-controlled trial to evaluate the effectiveness of online Community Reinforcement and Family Training (CRAFT) on the wellbeing of family members with a relative experiencing substance dependence and mental illness
Source: BMC Psychiatry. 2024 Jan 10;24:43. doi: 10.1186/s12888-023-05487-0 (PMC10782775; doi:10.1186/s12888-023-05487-0)

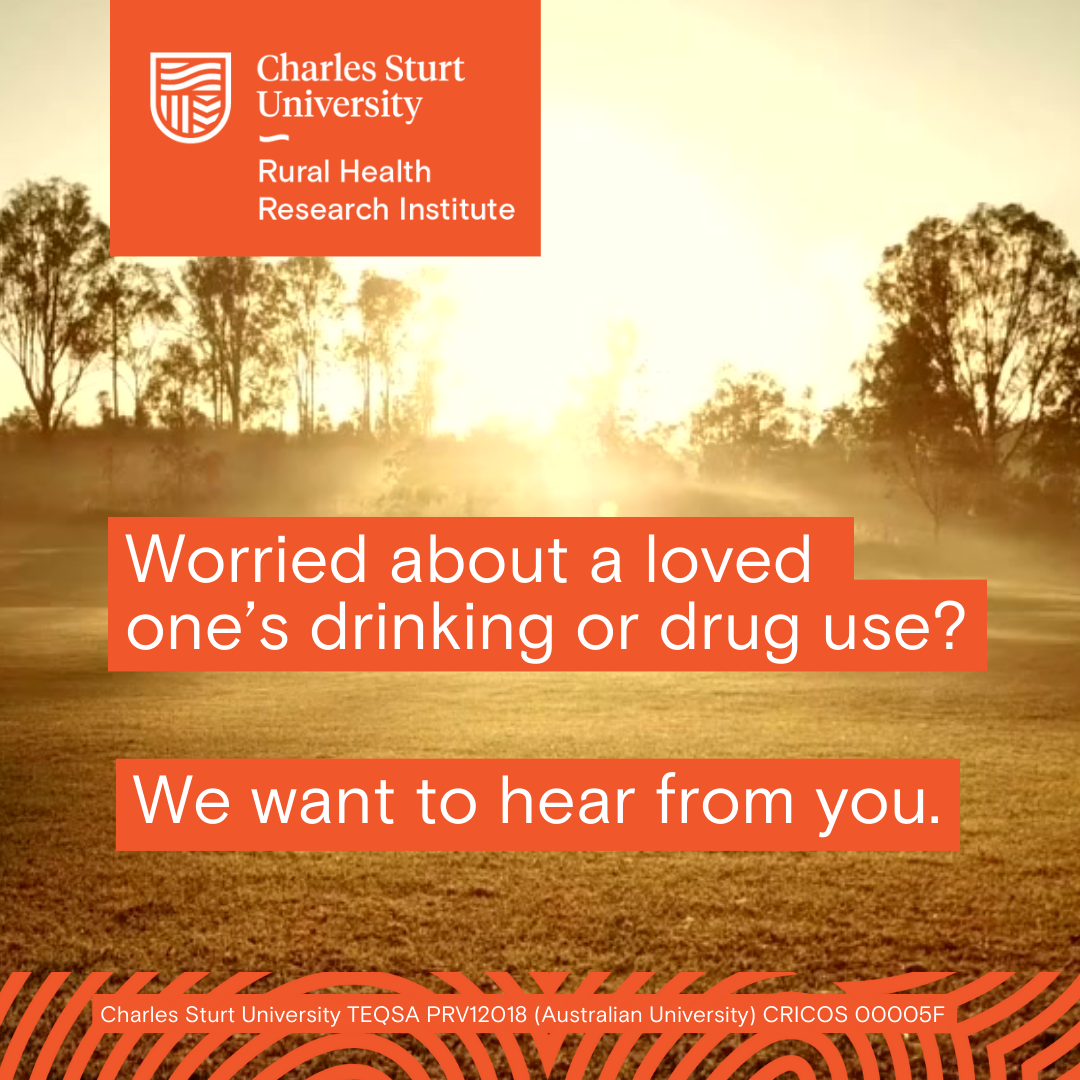

Supplement: Supplementary file 2 — Additional file 2. [file 12888_2023_5487_MOESM2_ESM.png]
