# Supplementary material for: Study protocol for the family empowerment program: a randomized waitlist-controlled trial to evaluate the effectiveness of online Community Reinforcement and Family Training (CRAFT) on the wellbeing of family members with a relative experiencing substance dependence and mental illness
Source: BMC Psychiatry. 2024 Jan 10;24:43. doi: 10.1186/s12888-023-05487-0 (PMC10782775; doi:10.1186/s12888-023-05487-0)
Supplement: Supplementary file 3 — Additional file 3. [file 12888_2023_5487_MOESM3_ESM.pdf]

Family Empowerment Program Session SUMMARY

This form is to be returned to the research team

Please complete for each consenting participant

If there is more than one family member participating - one form per family member

N.B. Please only include the activities that were completed with the participant

---

This information is used to anonymously match each client – please use the same details for each participant

I__I__I<br>Participant's first and last initials (family member or concerned other)	I__I__I__I__I<br>Participant's year of birth	I__I__I<br>First and last initials of the relative (person misusing drugs or alcohol)	I__I__I<br>Number of people in the family participating in the program	I__I__I__I__I__I<br>Clinician Initials and year of birth

FEP ACTIVITY		**Date:** (Please enter each date you did the activity with the participant).	**Length of session (minutes):**	**Action plan for the week**
	1. Introduction to the program, building motivation and self-care, Happiness Scale and goal setting			
	2. Positive Communication *Milestone conversation 1*: What is most important to your relative?			
	3. Functional analysis: typical episode of relative's drinking/using behaviour *Milestone conversation 2*: What benefits does your relative get from substance use?			
	4. Positive reinforcement of healthy behaviours including Functional analysis of healthy behaviours (non-drinking/using behaviours)			
	5. Problem solving & Natural consequences *Milestone conversation 3:* How does your relative feel about treatment?			
	6. Engaging relative into treatment & Program review - planning to maintain progress. Additional sessions?			

Online delivery methods

How would you rate the **sound/video** quality?	How would you rate the **ease of use**?
Week 1. (poor) 1_____2_____3_____4_____5 (excellent)<br>Week 2. (poor) 1_____2_____3_____4_____5 (excellent)<br>Week 3. (poor) 1_____2_____3_____4_____5 (excellent)<br>Week 4. (poor) 1_____2_____3_____4_____5 (excellent)<br>Week 5. (poor) 1_____2_____3_____4_____5 (excellent)<br>Week 6. (poor) 1_____2_____3_____4_____5 (excellent)	Week 1. (poor) 1_____2_____3_____4_____5 (excellent)<br>Week 2. (poor) 1_____2_____3_____4_____5 (excellent)<br>Week 3. (poor) 1_____2_____3_____4_____5 (excellent)<br>Week 4. (poor) 1_____2_____3_____4_____5 (excellent)<br>Week 5. (poor) 1_____2_____3_____4_____5 (excellent)<br>Week 6. (poor) 1_____2_____3_____4_____5 (excellent)

Did you experience any technical challenges?

Week 1. Yes No (circle)

Week 2. Yes No

Week 3. Yes No

Week 4. Yes No

Week 5. Yes No

Week 6. Yes No

Do you have any additional comments about the session for this week?

Week 1. ____

Week 2. ____

Week 3. ____

Week 4. ____

Week 5. ____

Week 6. ____

Optional sessions: dates and discussion topics

